# Advances in Drug Discovery of New Antitubercular Multidrug-Resistant Compounds †

**DOI:** 10.3390/ph10020051

**Published:** 2017-06-01

**Authors:** Guilherme Felipe dos Santos Fernandes, Chung Man Chin, Jean Leandro Dos Santos

**Affiliations:** 1 Institute of Chemistry, São Paulo State University (UNESP), Araraquara 14800060, Brazil; guilhermefelipe@outlook.com; 2 School of Pharmaceutical Sciences, São Paulo State University (UNESP), Araraquara 14800903, Brazil; chungmanchin@gmail.com

**Keywords:** tuberculosis, drug discovery, antitubercular compounds, multidrug-resistant tuberculosis

## Abstract

Tuberculosis (TB), a disease caused mainly by the *Mycobacterium tuberculosis (Mtb)*, is according to the World Health Organization (WHO) the infectious disease responsible for the highest number of deaths worldwide. The increased number of multidrug-resistant (MDR-TB) and extensively drug-resistant (XDR-TB) strains, and the ineffectiveness of the current treatment against latent tuberculosis are challenges to be overcome in the coming years. The scenario of drug discovery becomes alarming when it is considered that the number of new drugs does not increase proportionally to the emergence of drug resistance. In this review, we will demonstrate the current advances in antitubercular drug discovery, focusing on the research of compounds with potent antituberculosis activity against MDR-TB strains. Herein, active compounds against MDR-TB with minimum inhibitory concentrations (MICs) less than 11 µM and low toxicity published in the last 4 years in the databases PubMed, Web of Science and Scopus will be presented and discussed.

## 1. Introduction

According to the World Health Organization (WHO), tuberculosis (TB) is the infectious disease responsible for the highest number of deaths worldwide, surpassing even the number of deaths caused by the human immunodeficiency virus (HIV). The last surveys conducted by WHO pointed out 9.6 million new cases of the disease and 2 million deaths in 2015 [1]. In addition, the emergence and increase of multidrug-resistant (MDR; defined as resistant as a minimum to rifampicin (RMP) and isoniazid (INH)) and extensively drug-resistant (XDR; defined as MDR plus additional resistance to at least one fluoroquinolone and one second-line injectable drug) strains of *Mycobacterium tuberculosis* (*Mtb*) have been alarming authorities around the world. These tuberculosis strains presents low cure rates and high mortality rates due to difficulties in the treatment [2,3]. Furthermore, cases of totally drug-resistant tuberculosis (TDR-TB) have been reported in the clinic [4,5].

Over the last few years, progress has been made in the search for new anti-TB compounds [6,7]. The current drug pipeline shows ten drug candidates under preclinical and early phase 1 development for drug-sensitive and/or drug-resistant tuberculosis. Regarding MDR-TB, the drug pipeline presents six compounds in phase 2 and 3 trials. Bedaquiline (Sirturo^®^, Janssen Therapeutics, Titusville, NJ, USA) and delamanid (Deltyba^®^, Otsuka Pharmaceutical, Tokyo, Japan) remain in trial phase 3; despite their approval in several countries justified by the emergency caused by MDR-TB. Sutezolid and pretomanid are, in phase 2 and 3 trials, respectively. The repurposed drugs clofazimine and levofloxacin are in phase 3 and 2 trials, respectively [8,9].

Despite the recent advances, strains resistant to these new molecules have already been reported [10,11,12] reinforcing the urgent need to develop novel drugs for tuberculosis treatment. Indeed, the research of new drugs against TB plays a crucial role in reducing the incidence and mortality necessary to achieve global worldwide goals established by the WHO [1].

Therefore, this review article aims to create new perspectives to design and develop new anti-TB drugs against multi-drug resistant strains. We will focus on the challenges and advances in antitubercular drug discovery involving MDR-TB. In order to describe those most promising compounds, we included here only active compounds against MDR-TB with minimum inhibitory concentrations (MICs) inferior to 11 µM and low toxicity published in the last 4 years in the following databases: PubMed, Web of Science and Scopus. All MIC values against MDR-TB strains presented herein were converted to µM in order to establish a comparison among the compounds.

## 2. Antituberculosis Compounds

**Figure 1:**
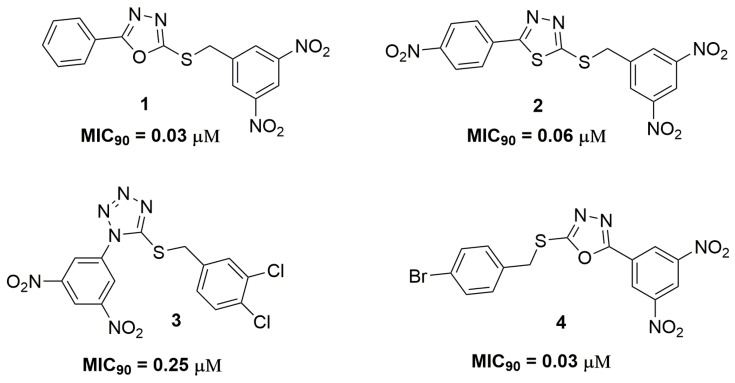
Nitro-containing compounds with antituberculosis activity. MIC: minimum inhibitory concentration.

Roh and coworkers reported a series of 3,5-dinitrobenzylsulfanyl-1,3,4-oxadiazoles and thiadiazoles derivatives with potent activity against replicating and nonreplicating *Mycobacterium tuberculosis*. Compounds (**1**) and (**2**) (Figure 1) were the most promising in this series and presented MIC_90_ values against several MDR strains below 0.25 μM. Moreover, these compounds have been shown to be active against dormant mycobacteria using a luciferase assay. In vitro cytotoxicity studies against mammalian cell lines and isolated human hepatocytes showed low toxicity with inhibitory concentration (IC_50_) values above 20 μM. Both compounds also did not exhibit mutagenic activity and activity against several bacteria and fungi. Furthermore, studies were performed which attempted to characterize the possible target of the compounds. In vitro studies suggested that compounds (**1**) and (**2**) could act inhibiting the synthesis of nucleic acids [13]. The same research group has reported a new series of 3,5-dinitrophenyl 1,3,4-oxadiazole-2-thiols and tetrazole-5-thiols as promising antitubercular agents. They have discovered compounds (**3**) and (**4**) (Figure 1) as highly potent and selective derivatives against seven MDR and XDR strains with MIC_90_ values below 0.5 μM. In addition, these compounds exhibited low cytotoxicity against human hepatocellular carcinoma (HuH7), human epidermoid carcinoma (A431), Madin-Darby canine kidney cells (MDCK), human hepatocellular carcinoma (HepG2) cell lines, with IC_50_ above 30 μM [14].

**Figure 2:**
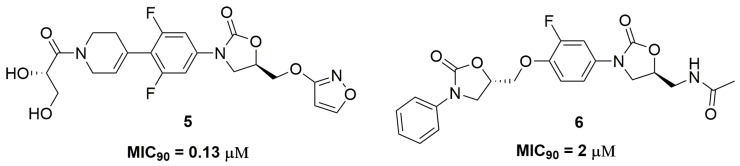
Oxazolidinone derivatives as antitubercular agents.

Oxazolidinone is an important class of antibiotic used for treatment of infections caused by Gram-positive pathogens, being the drug linezolid the main representative of this class [15]. This scaffold acts through the inhibition of protein synthesis by binding to the 50S ribosomal subunit and blocking the binding of transfer RNA (tRNA) [16]. Researchers from AstraZeneca^®^ (AstraZeneca R&D, Bangalore, India) have identified an oxazolidinone derivative, namely AZD5847 (**5**) (Figure 2), with outstanding antitubercular activity against a panel of several clinical isolates of *Mtb* with different resistance profiles, including single drug-resistant strains. AZD5847 showed improved extracellular and intracellular activity compared to linezolid, exhibiting MIC_90_ values in a range of 0.13 and 1 μM in the tested strains and a reduction of 1-log-unit in macrophage intracellular bacilli. Furthermore, the authors also demonstrated that this compound (**5**) also acts through the inhibition of protein synthesis [17]. AZD5847 was clinically tested as an antimycobacterial drug in a phase 2; however, several adverse effects were observed, including serious hepatic and haematological toxicities [18]. Wei and coworkers also reported a series of bis-oxazolidinone derivatives with antitubercular activity. Compound (**6**) (Figure 2) exhibited MIC_90_ values at values ranging from 2 to 8 μM against several clinical MDR and XDR-TB strains. In addition, the authors demonstrated the low cytotoxicity of this compound against monkey fibroblast-like kidney cell (VERO) with IC_50_ above 5000 μM and high selectivity against mycobacteria [19].

**Figure 3:**
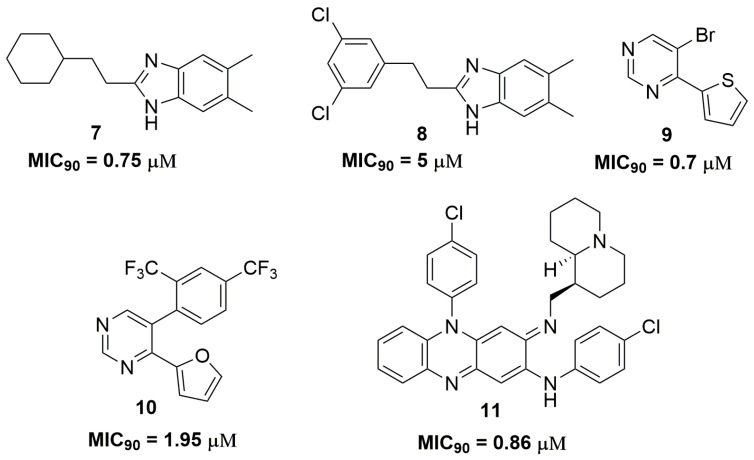
Nitrogen-containing heterocycles with antituberculosis activity.

The antitubercular activities of several nitrogen heterocyclic compounds have been extensively published in the scientific literature. For instance, a series of 1*H*-benzo[*d*]imidazole derivatives have been reported to possess promising activity against a clinically isolated strain resistant to *p*-aminosalicylic acid (PAS), INH, ethambutol (ETB) and RMP. Compound (**7**) (Figure 3) showed a MIC_90_ value of 0.75 μM against this wild strain and low cytotoxicity against human non-small lung cancer (A549) and pig kidney epithelial cell line (LLC-PK1) cell lines, with IC_50_ values of 11.15 and 43.94 μM, respectively [20]. Later, the same authors published a novel series of 1*H*-benzo[*d*]imidazoles derivatives. Nevertheless, this new series exhibited worse activity than compound (**7**). The best compound (**8**) (Figure 3) showed an MIC_90_ value of 5 μM in the same MDR strain and IC_50_ values of 58 and 7.8 μM against neonatal human dermal fibroblasts (HDFn) and human epidermal keratinocyte progenitors (HEK) cell lines, respectively [21].

Charushin and coworkers have synthesized a series of fifteen pyrimidine derivatives and evaluated their antituberculosis activity against a clinical isolated MDR-TB strain resistant to RMP and INH. Compound (**9**) (Figure 3) has shown the most promising activity with MIC_90_ of 0.7 μM. Mice acute toxicity revealed a median lethal dose (LD_50_) of 315 mg/kg for this molecule [22]. Additional studies performed by this same research group have described other pyrimidine derivatives with similar antituberculosis activity. The most promising compound (**10**) (Figure 3) exhibited an MIC_90_ value of 1.95 μM against a clinically isolated MDR-TB strain and LD_50_ of 600 mg/kg in acute toxicity using mice [23]. Quinolizidine-related compounds have also been reported with antituberculosis activity in a study involving fifteen derivatives. The series was evaluated against several MDR-TB strains. Compound (**11**) (Figure 3) was the most active in the series with MIC_90_ of 0.86 μM. In addition, this compound exhibited low cytotoxicity against VERO cells with IC_50_ of 68 μM. [24].

**Figure 4:**
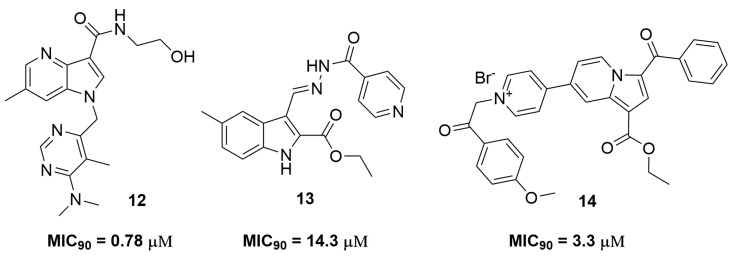
Nitrogen-containing fused heterocycles with potent activity against multidrug-resistant tuberculosis (MDR-TB).

Several nitrogen-containing fused heterocycles play an important role in the anti-TB Medicinal Chemistry. Shirude and coworkers [25] from AstraZeneca^®^ (AstraZeneca R&D, Bangalore, India) have reported a promising 1,4-azaindole derivative identified after an optimization process [26]. Compound (**12**) (Figure 4) was characterized as a potent derivative against MDR-TB, with MIC_90_ values ranging from 0.78 to 1.56 μM with low cytotoxicity against human leukemia monocytic (THP-1) cells (IC_50_ > 100 μM). In vivo studies were performed with compound (**12**) in order to determine its efficacy and pharmacokinetics profile. In rats, this molecule was able to reduce the bacterial burden on a logarithmic scale of 1 log_10_ colony forming units (CFUs)/lung at 300mg kg^−1^ of body weight, and statistically significant dose-dependentefficacy was observed. On the other hand, pharmacokinetic analysis revealed low clearance rates, excellent bioavailability and no interference with any of the cytochrome P450 (CYP450) isoenzymes. Nevertheless, this compound presented a rapid metabolism in the presence of mouse liver microsomes [25]. Unsuccessful attempts to optimize this compound have identified compound (**13**) as active against INH-resistant strains; however, with MIC_90_ value of 14.3 μM [27]. Danac and Mangalagiu have reported the synthesis and antituberculosis activity of a series of fused bipyridine heterocycles. Compound (**14**) (Figure 4) presented promising activity against several single resistant *Mtb* strains, with MIC_90_ values ranging from 3.3–9.2 μM [28].

**Figure 5:**
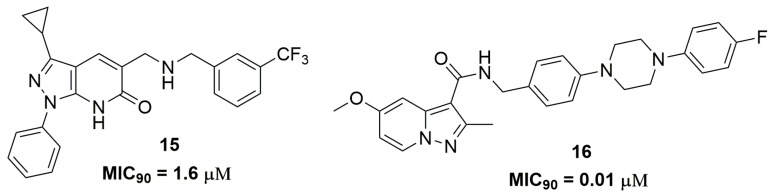
Compounds with antitubercular activity against MDR and extensively-drug resistant tuberculosis (XDR-TB).

Raichurkar and coworkers also from AstraZeneca^®^ (AstraZeneca R&D, Bangalore, India) have reported the discovery of pyrazolopyridones derivatives as promising antitubercular compounds [29]. Initially, they investigated the antituberculosis activity of these compounds against a panel of sensitive and clinical isolated single drug-resistant strains. Compound (**15**) (Figure 5) presented MIC_90_ values ranging from 1.6–3.1 μM and low cytotoxicity against the human A549 cell line (IC_50_ 160 μM). The authors also demonstrated through biochemical screening and genetic mapping that the target of compound (**15**) is decaprenylphosphoryl-*β*-_D_-ribose-2′-epimerase (DprE1), an enzyme that plays an important role in the biosynthesis of components of the mycobacterial cell wall [30,31]. In vitro drug metabolism and pharmacokinetics properties were performed for this compound, that exhibits distribution coefficient (log*D*) of 3.9 and suboptimal aqueous solubility (1 μM), free plasma protein binding (1%), and clearance (27 μL/min/kg) [29]. In another work, the synthesis and antituberculosis activity of pyrazolo[1,5-*a*]pyridine-3-carboxamide derivatives were reported. Specifically, compound (**16**) (Figure 5) has shown potent in vitro potency at nanomolar concentration, for which MIC_90_ values ranged from 11.1 to 223 nM against several clinically isolated MDR-TB strains. In addition, they evaluated the in vivo efficacy of compound (**16**) in a mouse model infected with the selectable marker-free autoluminescent *Mtb* H37Ra, a non-virulent strain. They observed a bactericidal activity of this compound, resulting in a reduction of bacterial burden using a modified real-time monitoring noninvasive bioluminescence assay [32].

**Figure 6:**
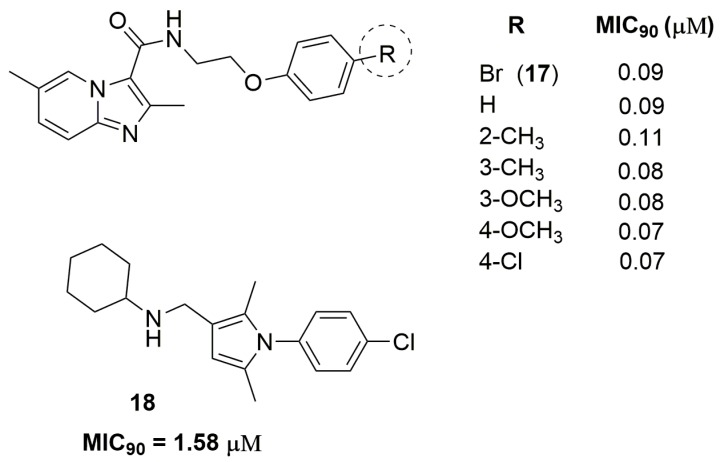
Compounds with antitubercular activity against MDR-TB.

Xuefu You and coworkers have identified a potent imidazo[1,2-*a*]pyridine derivative against two clinically isolated MDR strains resistant to INH and RMP. Compound (**17**) (Figure 6) has exhibited MIC_90_ values ranging from 0.09 to 0.13 μM and low in vivo acute toxicity in mice and rats. Furthermore, this compound exhibited acceptable pharmacokinetic properties, with maximum serum concentration (*C*_max_) of 225 ng/mL, elimination half-life (*T*_1/2_) of 1.5 h and clearance of 86,284 mL/h/kg [33]. Castagnolo and coworkers have reported a series of pyrrole derivatives designed as hybrids of the antitubercular drug candidates BM212 and SQ109. They obtained in the first generation a hybrid compound with MIC_90_ value of 17.8 μM against two MDR clinical isolates. After, using molecular simplification, the authors have discovered the second-generation of this hybrid derivative (compound **18**) (Figure 6) as a potent antitubercular agent with MIC_90_ value of 1.58 μM. Furthermore, this compound was able to inhibit the mycobacteria drug efflux pump at potency comparable to that of verapamil [34].

**Figure 7:**
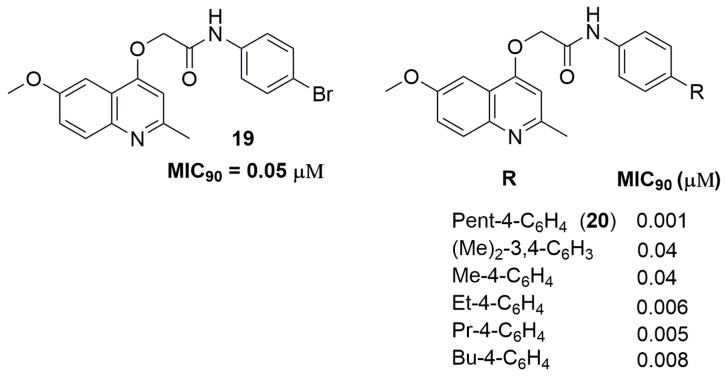
Quinoline derivatives with antituberculosis activity.

Quinoline and derivatives represent an important class of heterocyclic in the medicinal chemistry field because its wide range of biological activities, including antituberculosis [35]. Machado and coworkers have reported a series of quinoline derivatives with inhibitory activity against a clinical isolate of an MDR-TB strain. Compound (**19**) (Figure 7) showed a potent MIC_90_ value of 0.05 μM and activity against macrophage intracellular mycobacteria. Moreover, the authors also performed metabolic stability and drug–drug interaction studies. Compound (**19**) exhibited moderate metabolic stability in the human S9 fraction, with an in vitro intrinsic clearance (Cl_int_) of 14.3 mL/min/kg and *T*_1/2_ of 21.8 min. In addition, this derivative did not alter the enzymatic levels in the liver, suggesting a minimal risk of metabolic drug−drug interactions [36]. Later, the same group has reported a novel series of quinoline derivatives with improved antituberculosis activity. Compound (**20**) (Figure 7) exhibited a potent MIC_90_ value of 1 nM against a clinical isolate of an MDR-TB strain with a Cl_int_ of 14.8 mL/min/kg and *T*_1/2_ of 19.4 min [37].

**Figure 8:**
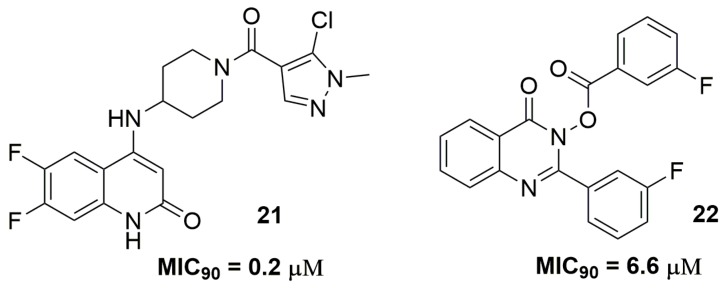
Compounds with antituberculosis activity.

Ghorpade and coworkers from AstraZeneca^®^ (AstraZeneca R&D, Bangalore, India) have discovered a quinolone derivative as a promising antituberculosis agent. The authors have assessed several properties of the lead compound (**21**) (Figure 8), including mechanism of action studies, in vitro/in vivo pharmacokinetics and drug metabolism. This quinoline derivative presented MIC_90_ values ranging from 0.2 to 3.12 μM against several single drug-resistant strains. The authors showed that the target of compound (**21**) is the mycobacterial DprE1 enzyme. In addition, in vivo pharmacokinetics studies pointed out compound (**21**) with *C*_max_ of 4.9 μM, plasma clearance of 34.4 mL/min/kg and *T*_1/2_ of 0.5 h [38]. A series of quinazolinone derivatives have been reported, showing potent antitubercular activity [39]. Compound (**22**) (Figure 8) exhibited MIC_90_ of 6.6 μM against several MDR and XDR-TB clinical isolates. In addition, the authors suggested that the *Mtb* acetohydroxy-acid synthase (AHAS) is the target of these quinazolinone derivatives [39]. AHAS is an enzyme that plays an important role in the branched-chain amino acids (BCAAs) biosynthetic pathway and its inhibition seems to be a potential target for anti-TB drugs [40].

**Figure 9:**
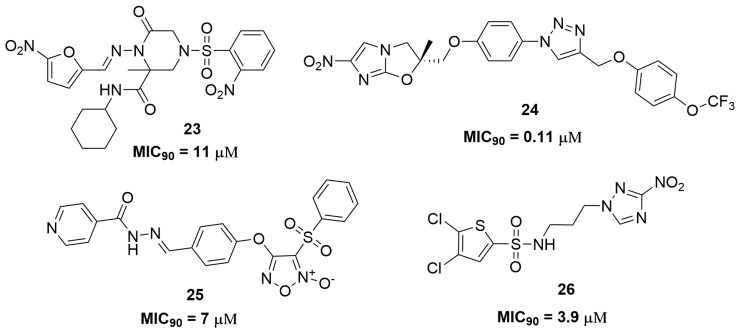
Compounds with antituberculosis activity that acts through the release of reactive oxygen and nitrogen species.

Compounds that acts through the release of reactive oxygen and nitrogen species, such as furoxan and nitro compounds, have been extensively explored as antituberculosis agents [41,42,43]. Brönstrup and coworkers have reported a series of nitrofuran derivatives with selective activity against *Mtb*. Specifically, compound (**23**) (Figure 9) exhibited MIC_90_ of 11 μM against two MDR-TB clinical isolates. The authors also evaluated the spectrum of activity for compound (**23**) and they did not find activity against a panel of Gram-positive and Gram-negative bacteria [44]. The nitroimidazole class is another important scaffold in the medical chemistry of antituberculosis agents, especially its main representative, the drug delamanid (Deltyba^®^, Otsuka Pharmaceutical) [45,46]. A series of nitroimidazole derivatives have been reported as presenting an outstanding antituberculosis activity against an MDR-TB clinical isolate. For instance, the most promising compound (**24**) (Figure 9) presented a MIC_90_ of 0.11 μM. The in vivo pharmacokinetics showed a good profile, with *C*_max_ of 0.54 μg/mL, *T*_1/2_ of 2 h and no CYP inhibition at three different concentrations tested (10, 30 and 100 μM). In vivo efficacy of compound (**24**) was assessed using a mice model for acute infection. Using an infected mice model, this compound was able to reduce the bacterial burden on a logarithmic scale of 1.8 log_10_ CFU at 100 mg kg^−1^ once daily for 28 days. [47]. Furoxan derivatives also have been reported showing its promising application as antitubercular compounds. Specifically, compound (**25**) (Figure 9) exhibited MIC_90_ of 7 μM against MDR-TB clinical isolates. Furthermore, the authors have demonstrated that this compound may act through the release of nitric oxide [48]. Smith and coworkers have discovered a nitrotriazole derivative as a promising antitubercular agent. Compound (**26**) (Figure 9) showed MIC_90_ values below 3.9 μM against several single drug-resistant strains. In addition, compound (**26**) exhibited intracellular activity against infected J774 macrophages, resulting in a reduction of 1.18 log of intracellular bacilli burden [49].

**Figure 10:**
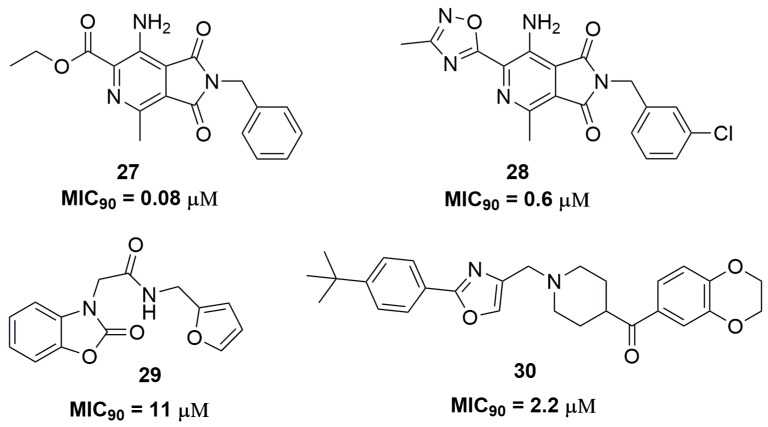
Heterocyclic compounds as promising agents for the treatment of MDR-TB.

Chibale and coworkers have identified the compound pyrrolo[3,4-*c*]pyridine-1,3(2*H*)-dione (**27**) (Figure 10) through a phenotypic screening with potent antituberculosis activity; however, this compound presented drawbacks regarding its metabolic stability. The optimization of this molecule leads to compound (**28**) (Figure 10), which demonstrated MIC_90_ values below 0.6 μM against several clinical isolates and good metabolic stability in liver microsomes. The mycobacterial respiratory cytochrome bc1 complex was identified as the target for these compounds. Nevertheless, mouse pharmacokinetics studies showed high clearance and low plasma exposure for compound (**28**) [50]. In another study, a series of twenty-seven benzo[*d*]oxazol-2(3*H*)-one derivatives were synthesized. Specifically, compound (**29**) (Figure 10) exhibited a promising antituberculosis activity against an XDR-TB clinical isolate, with MIC_90_ of 11.47 μM. The authors also demonstrated that compound (**29**) acts through the inhibition of the mycobacterial 2-trans-enoyl-acyl carrier protein reductase (InhA), a key enzyme involved in the mycolic acid biosynthesis in *Mtb* [51]. Shuyi Si and coworkers have discovered several disubstituted oxazole analogues as potent antituberculosis agents. Among the twenty-five compounds evaluated, compound (**30**) (Figure 10) showed promising activity with MIC_90_ values of 2.2 and 4.3 μM against XDR-TB and MDR-TB, respectively. Moreover, compound (**30**) proved to be selective for *Mtb* because no activity against other bacteria was observed [52].

**Figure 11:**
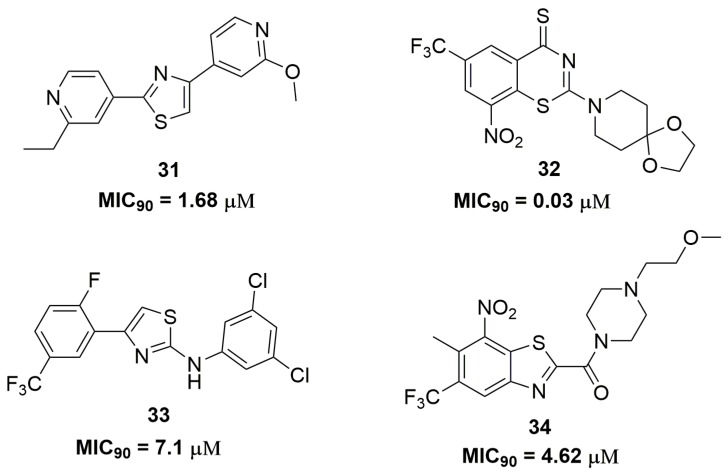
Sulfur-containing heterocycles with potent antitubercular activity.

Sulfur-containing heterocycles have been widely exploited as antituberculosis agents [53]. Researches from AstraZeneca^®^ (AstraZeneca R&D, Bangalore, India) have discovered a diarylthiazole derivative after molecular optimizations in a previously identified hit. Compound (**31**) (Figure 11) showed potent antituberculosis activity with MIC_90_ values below 1.68 μM against several MDR-TB clinical isolates. In vitro drug metabolism and pharmacokinetics parameters were assessed for compound (**31**), which exhibited solubility of 31 μM, mouse plasma protein binding of 17% and mouse clearance of 170.4 μL/min/mg. Moreover, the authors have characterized the PrrBA as the molecular target of this derivative [54]. PrrBA is a two-component system composed of the PrrB histidine kinase and PrrA response regulator and it plays an important role in mycobacterial virulence and metabolic adaptation to stress [55,56]. In another work, Luoting Yu and coworkers reported a benzothiazinethione derivative as a potent antitubercular agent [57]. Compound (**32**) (Figure 11), an analog of the drug candidate BTZ043 [58], presented MIC_90_ values below 0.03 μM against several MDR and XDR-TB clinical isolates. The pharmacokinetics in rats of this compound showed *C*_max_ of 193 ng/mL, *T*_1/2_ of 1.45 h and 44.4% of absolute oral bioavailability. Furthermore, compound (**32**) demonstrated in vivo efficacy in a mice model, which was able to reduce the *Mtb* burden in lungs by 3.4 logs CFU [57]. Thiazole and its analogue benzothiazole are also important sulfur-containing heterocycles with antituberculosis activity. For instance, the synthesis of a series of thirty-four thiazole derivatives have been reported and their antitubercular activity was assessed against several single drug-resistant strains. Compound (**33**) (Figure 11) showed MIC_90_ values ranging from 7.1 and 12.0 μM [59]. Ramachandran and coworkers at AstraZeneca^®^ (AstraZeneca R&D, Bangalore, India) also reported benzothiazoles derivatives as antitubercular. They discovered compound (**34**) (Figure 11) as a promising antituberculosis agent with MIC_90_ values below 4.62 μM against several single drug-resistant isolates. DprE1 was characterized as the target of compound (**34**). In addition, this compound demonstrated safety profile with low cytotoxicity against human A549 cell line (IC_50_ > 100 μM), negative Ames assay and moderate CYP isoform inhibition [60].

**Figure 12:**
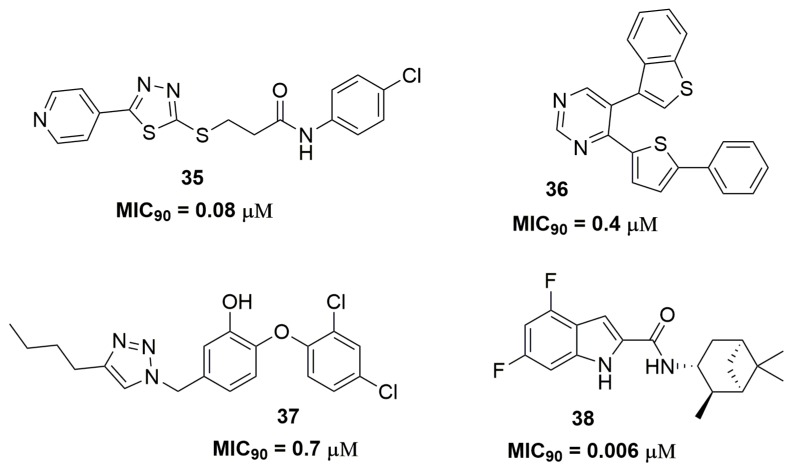
Sulfur-containing compounds, triazole and indole as potent anti-TB agents.

Mahajan and Dhawale reported the synthesis of a series of thiadiazoles derivatives as promising candidates for resistant tuberculosis treatment. Among the thirty-three compounds synthesized, compound (**35**) (Figure 12) showed the best antimycobacterial activity with MIC_90_ values ranging from 0.08 to 0.66 μM against several MDR and XDR-TB clinical isolates [61]. Thienyl-substituted pyrimidines derivatives also have been reported with potent activity against an MDR-TB strain. Specifically, compound (**36**) (Figure 12) demonstrated MIC_90_ of 0.4 μM and mouse LD_50_ of 45 mg/kg [62]. In another study, Kozikowski and coworkers synthesized a series of triclosan derivatives with the mycobacterial InhA enzyme characterized as the molecular target. Compound (**37**) (Figure 12) exhibited MIC_90_ of 0.7 μM against two MDR-TB strains [63]. Later, the same research group identified an indole-2-carboxamide derivative designed from a structure-activity relationship analysis of previously obtained compounds. Compound (**38**) (Figure 12) showed outstanding antituberculosis activity against MDR and XDR-TB strains. The MIC_90_ values for these strains ranged from 0.006 to 0.047 μM. Pharmacokinetics results showed a *C*_max_ of 1.71 μg/mL, *T*_max_ of 4 h, low inhibition in the tested CYP isoforms (<40%) and negative results in the bacterial reverse mutation assay in *Salmonella typhimurium* tester strains. Moreover, the in vivo efficacy of this compound was assessed in a mouse infection model and it was able to reduce the bacterial burden on a logarithmic scale of 2.12 log_10_ CFU at 100 mg kg^−1^ dosage level in the lungs after 4 weeks of treatment protecting the mice from death. Altogether, these results pointed out compound (**38**) as a promising drug candidate for human clinical trials [64].

**Figure 13:**
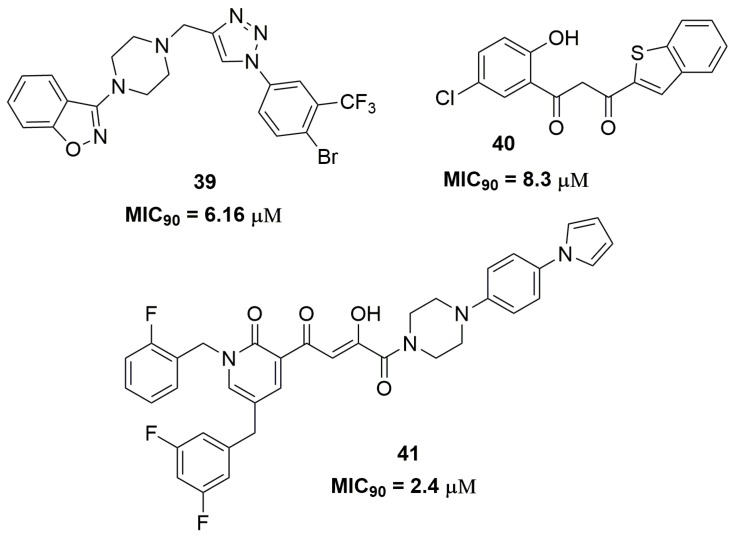
Miscellaneous of compounds with activity against MDR-TB.

Indeed, the scientific literature presents several works involving only the synthesis and phenotypic screening of compounds against MDR strains accompanied by in silico studies. Among these compounds, several heterocycles and scaffolds are contemplated, including benzo[*d*]isoxazole (**39**) [65], benzo[*b*]thiophenes (**40**) [66], disubstituted piperazine (**41**) [67], and benzoxazole (**42**) [68] (Figure 13).

For instance, compound (**39**) exhibited MIC_90_ of 6.16 μM against an MDR-TB isolate and low cytotoxicity against mouse macrophage (RAW264.7) cell lines with IC_50_ of 222.92 μM. In addition, the authors performed docking studies on the mycobacterial pantothenate synthetase enzyme and compound (**39**) showed a good interaction in the enzyme active site with docking score of −9.2 kcal/mol [65]. This enzyme is involved in the biosynthesis of coenzyme A and acyl carrier protein [69]. The benzo[*b*]thiophene (**40**) also exhibited potent antituberculosis activity against a multidrug-resistant strain with MIC_90_ of 8.3 μM. Docking studies on the DprE1 enzyme were performed and compound (**40**) showed a docking score of −8.7 kcal/mol, suggesting that this enzyme could be the potential molecular target [66]. Likewise, compound (**41**) showed a promising antitubercular activity against an MDR-TB strain with MIC_90_ of 2.4 μM. In vitro microsome stability study exhibited a *T*_1/2_ of 14.4 h and low inhibition of two isoforms of cytochrome P450, namely CYP3A4 and CYP2D6. Moreover, the authors suggested that compound (**41**) might act through inhibition of mycobacterial DNA-dependent RNA polymerase [67]. The benzoxazole derivative (**42**) also showed to be a promising antituberculosis agent. The MIC_90_ evaluated for this compound was 3.2 μM against XDR-TB strains and it demonstrated in vivo activity similar to that of RMP in a mouse model infected with the selectable marker-free autoluminescent *Mtb* strain H37Ra [68].

The drug discovery process is highly complex and involves several steps. Among these steps, the determination of the physicochemical properties of potentially active compounds plays an important role in the initial stages [70,71]. Indeed, some authors suggest that bioactive compounds with adequate physicochemical properties should be prioritized rather than highly active compounds with inadequate physicochemical properties [72]. Currently, several computational programs predict these properties based on databases of compounds and mathematical formulas [73,74]. Therefore, we subjected the most active compounds described in this review with MIC_90_ values below 0.1 μM to an analysis of the Lipinski’s rule, which indicates whether a molecule could be an orally active drug in humans [75,76]. We calculated theoretical partition coefficient (cLog*P*) values, molecular weight and number of hydrogen bond donors and acceptors using the software OSIRIS DataWarrior (Table 1). The data showed that the majority of the compounds presented adequate properties according to the Lipinski’s rule. In addition, the software also predicted the drug-likeness of these compounds. Briefly, this drug property indicates whether a compound contains fragments that are frequently present in commercial drugs. The most active compounds selected presented values of drug-likeness in a range of −14.43 to 7.03. The majority of marketed drugs show values between −5 and 5 [77].

**Figure 14:**
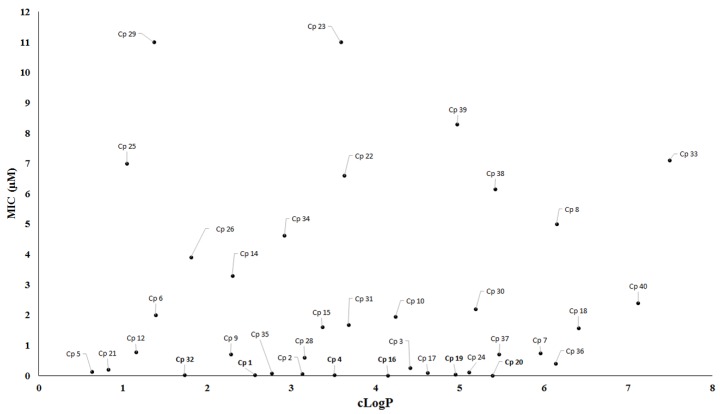
Correlation between MIC values and cLog*P* for the compounds. cLogP: calculated partition coefficient.

During drug development, lipophilicity is the most important physicochemical property that might be analyzed for tuberculosis drug discovery because this property can affect the solubility, permeability and bioavailability of compounds [78]. Furthermore, lipophilicity is recognized to impact on a number of drug-like characteristics including pharmacokinetics and toxicology properties [79,80]. For instance, compounds with high lipophilicity have been related to hepatotoxicity and undesirable non-specific interactions [81,82]. Nevertheless, highly lipophilic compounds should not be discarded in screening programs for antituberculosis drugs. One example is the drug bedaquiline, which has a cLog*P* value of 7.3. Likewise, low lipophilicity is not an impediment to tuberculosis drug discovery, considering the number of approved drugs with low lipophilicity, including isoniazid (cLogP = −0.67), ethambutol (cLogP = 0.12), pyrazinamide (cLogP = −0.68), kanamycin (cLogP = −5.2) and cycloserine (cLogP = −1.2). Therefore, we have evaluated the relationship between MIC values and cLog*P* (Figure 14). The data showed that the majority of active compounds described in this review presented cLog*P* values in a range from 2 to 6.

## 3. Conclusions

Phenotypic screening seems to be a more promising approach to identify compounds active against MDR-TB than the target-based approach. Despite this, the search for new compounds active against resistant-TB remains a challenge. The molecular mechanism involved in the resistance and its possible targets is still not completely understood. However, even in this complicated scenario, in recent years, active compounds against resistant strains have been found at nanomolar concentrations. In addition, in vivo studies have shown that some of these compounds exhibited adequate pharmacokinetics for investigation in future studies. Additional efforts must be made in order to create strong networks worldwide to discover new drugs against this terrible disease.

## Figures and Tables

**Table 1:** MIC_90_ values and calculated physicochemical parameters.**^1^**

Compound	MIC_90_ (μM) ^2^	cLog*P* ^3^	H bond Donors	H bond Acceptors	Molecular Weight	Drug-Likeness
**1**	0.03	1.84	0	9	358.33	−4.43
**2**	0.06	1.22	0	11	419.39	−3.14
**4**	0.03	2.57	0	9	437.22	−6.22
**16**	0.01	2.83	1	7	473.55	7.03
**17**	0.09	2.98	1	5	388.26	1.53
**19**	0.05	3.75	1	5	401.25	−1.04
**20**	0.001	4.23	1	5	364.44	−1.44
**32**	0.03	1.23	0	7	433.43	−14.4
**35**	0.08	3.44	1	5	376.89	3.49
**38**	0.006	3.72	2	3	332.39	−1.2

^1^ Theoretical calculated values using OSIRIS DataWarrior program; ^2^ Minimum inhibitory concentration; ^3^ Calculated partition coefficient.
